# New Therapeutic Approaches against Inflammation and Oxidative Stress in Neurodegeneration

**DOI:** 10.1155/2022/9824350

**Published:** 2022-05-19

**Authors:** Erica Buoso, Fabrizio Biundo, Alessandro Attanzio

**Affiliations:** ^1^Department of Drug Sciences, University of Pavia, Via Taramelli 12/14, 27100 Pavia, Italy; ^2^Department of Developmental and Molecular Biology, Albert Einstein College of Medicine, 1300 Morris Park Ave, Bronx, NY 10012, USA; ^3^Department of Biological, Chemical and Pharmaceutical Sciences and Technologies (STEBICEF), University of Palermo, Via Archirafi 28, 90123 Palermo, Italy

Due to the increase of life expectancy, the world aging population has been increasing significantly and is expected to triple by 2050. Aging is a physiological change characterized by a progressive decline of biological functions and of the organism's ability to adapt to metabolic stress, and it is considered one of the main factors for neurodegenerative diseases [1]. Among these, Alzheimer's disease (AD) is the most common neurodegenerative pathology characterized by memory decline and dementia [2]. Markers of oxidative stress have been observed in brains of patients affected by amnestic Mild Cognitive Impairment (aMCI), and of late AD patients [3, 4]. Accumulation of oxidized molecules is caused by an imbalance between the production of reactive oxygen/nitrogen species (ROS/RNS) and the antioxidative cell systems, and has been indicated as one of the molecular events that characterize the pathogenesis of multiple neurodegenerative diseases [5]. In addition to oxidative stress, dysregulated chronic neuroinflammation has been described to be a driving force of decade-long neurodegenerative processes [6]. A large body of studies in the last decades detected an association between these two pathogenic events in brains of patients affected by neurodegeneration and in mouse models of various neurodegenerative diseases [7–9]. Hence, limiting these two intertwined pathologic factors may have greater efficacy for the treatment of neurodegenerative diseases [10].

This special issue collected a set of 7 multidisciplinary studies addressing the function of molecular mechanisms underlying the production of reactive oxidation and inflammation in different *in vitro* and *in vivo* models, and the potential therapeutic role of natural antioxidants.

The study conducted by Kent et al. focused on the role of the mitochondrial permeability transition pore (mPTP) which is a protein involved in ROS expansion [11]. mPTP opening is also associated with chronic inflammation and can be controlled by nicotinamide adenine dinucleotide (NAD+), an antioxidant agent declining with age [12]. The authors covered a large part of literature describing the role of mPTP in neurodegenerative diseases, particularly on Parkinson's disease (PD) and Alzheimer's disease (AD). Both AD and PD are associated with an increased oxidative damage of DNA, both of which are linked to mPTP opening and consequent ROS release [13]. Targeted therapies aiming at reducing the frequency and duration of mPTP opening may therefore be a promising path for the development of specific drugs against age-related declines of the central nervous system.

Lin and colleagues dissected the pathway leading to carbon monoxide releasing molecule-3 (CORM-3)-induced upregulation of heme oxygenases-1 (HO-1), a key enzyme that plays an important role in maintaining cellular homeostasis, in rat brain astrocytes (RBA-1) [14]. They observed that CORM-3-induced HO-1 expression was mediated through ROS generation by NADPH oxidase (Nox), and a mitochondria/ROS-dependent PI3K/Akt/mTOR cascade triggering FoxO1. The authors concluded that in RBA-1 cells, CORM-3-induced HO-1 expression is, at least partially, mediated through Nox, and their findings strengthened the previous observations indicating HO-1 potentiation as a potential therapeutic target [15].

In the experimental study conducted by Moon et al., calcineurin, a calcium-related protein phosphatase of type 2B expressed in the brain, was found to act as a critical checkpoint in the prion-dependent control of different cellular functions [16]. PrPsc can accumulate in the brain in pathological conditions and induce mitochondrial dysfunctions and reactive oxygen species (ROS) generation in neurons [17]. The authors observed that the human prion peptide increases mitochondrial ROS by activating calcineurin, and that calcineurin inhibition prevented the mitochondrial dysfunction and neuronal apoptosis induced by PrPsc. These results suggest that calcineurin plays a role in PrPsc-induced ROS release and neuronal apoptosis, and indicate it as another potential pharmacological target.

The systematic review of literature conducted by Adeyemi et al. focused on implications and therapeutic prospects of hypoxia and the kynurenine pathway in Alzheimer's disease (AD) [18]. Although the underlying molecular events or mechanisms connecting hypoxia to neurodegeneration are not well-understood, hypoxia-inducible factor 1-alpha (HIF-1*α*) is a master regulator of the cellular/tissue response to hypoxia and seems to be correlated with the pathogenesis of different neurodegenerative diseases, including AD [19]. The authors focused on HIF-1*α* role and hypoxia in the progression of AD underlining in a simple and schematic approach HIF-1*α* and kynurenine pathway and the possible connection between these two signaling cascades. This work indicated that hypoxia is related to oxidative stress and inflammation, which in turn affect tryptophane catabolism through indoleamine 2,3-dioxigenase (IDO) enzyme, resulting in neurotoxic metabolites that contribute to neurodegeneration. Despite the significant achievements in this field, the authors suggest that further *in vitro* and *in vivo* experiments are necessary to fully understand hypoxia and IDO roles in the kynurenine pathway in order to identify novel therapeutic targets.

The study conducted by Alvi et al. in pentylenetetrazole (PTZ)-kindled epileptic rats focused on carveol, a natural compound that possesses robust antioxidant, anti-inflammatory, and protective properties in various degenerative models [20]. It has been reported that oxidative stress can exacerbate epilepsy and the degree of oxidative damage is proportional to epileptic episodes [21]. Hence, the aim of this work was to investigate the effective dose of carveol, its mechanism of action in regulating Nrf2, and ultimately its neuroprotective effects. The authors reported that PTZ-kindled animals experienced oxidative stress and revealed diminished levels of superoxide dismutase (SOD), catalase (CAT), glutathione-S-transferase (GST), and glutathione (GSH) associated with elevated lipid peroxidation (LPO) and inflammatory cytokines level such as tumor necrosis factor-alpha (TNF-*α*), and mediators like cyclooxygenase (COX-2), and nuclear factor kappa B (NF*κ*B). Carveol was demonstrated to increase these antioxidants and reduced LPO levels together with a positive modulation of the antioxidant gene Nrf2 and its downstream target HO-1. Moreover, Nrf2 pathway activation inhibited proinflammatory cytokine release and downregulated the p-NF-*κ*B pathway, highlighting the anti-inflammatory potential of carveol. The strong involvement of Nrf2 pathway in the cytoprotective nature of carveol was confirmed by all-trans retinoic acid (ATRA) treatment, which abolished carveol effects and exacerbated PTZ toxicity. Furthermore, different studies have suggested that local immune response and inflammation are associated with the upregulation of hippocampal acetylcholinesterase (AChE) levels, resulting in cholinergic imbalance and epileptogenesis. In this context, the authors also reported that increased brain AChE level was significantly inhibited by carveol treatment, indicating a modulating effect of carveol on cholinergic transmission that is further linked to attenuated neuroinflammatory cascade. Additionally, carveol treatment was also found to ameliorate VEGF expression, indicating an improvement in PTZ-mediated angiogenesis. Altogether, these findings suggest that carveol, acting as a Nrf2 activator, attenuates seizure severity and neuroinflammation in PTZ-kindled epileptic rats.

Extensive research over the last few years has demonstrated the potential neuroprotective role of phytochemicals and their beneficial effects on the prevention of neurodegenerative diseases including Parkinson's disease. Epidemiological studies have shown that a diet based on the consumption of beverages such as tea, coffee, fruit, and vegetables is associated with a reduced risk of neurological damage or pathologically related diseases [22].

Balakrishnan et al. performed a comprehensive evaluation of various phytochemicals present in foods such as chrysin, vanillin, ferulic acid, thymoquinone, ellagic acid, caffeic acid, epigallocatechin-3-gallate, theaflavin, and other plant-derived antioxidant phytochemicals highlighting their beneficial and neuroprotective effects in different experimental models. In light of this, dietary antioxidant and anti-inflammatory phytochemicals or extracts from waste products of the food industry could be a potential new therapeutic strategy against the symptoms or progression of neurodegenerative diseases [23].

In line with this topic, Angeloni et al. provided an experimental study detecting the phenolic profile and the antioxidant and anti-inflammatory activity of spent coffee ground (SCG) extracts in cellular models of neuroinflammation. The main SCG components—caffeine, 5-O-caffeoylquinic acid, 3-O-caffeoylquinic acid, and 3,5-O-dicaffeoylquinic acid—proved to be efficient in counteracting oxidative stress and neuroinflammation *in vitro* by upregulating endogenous antioxidant enzymes such as thioredoxin reductase, heme oxygenase 1, NADPH quinone oxidoreductase, and glutathione reductase. Based on these findings, SCG extracts could represent a valuable source of potential neuroprotective bioactive molecules for the treatment of neurodegeneration [24].
